# Triple pathology in patients with temporal lobe epilepsy: A case report and review of the literature

**DOI:** 10.3892/etm.2013.1228

**Published:** 2013-07-19

**Authors:** KANG YANG, JING SU, ZENGCHUN HU, RUI LANG, XU SUN, XINYU LI, DONG WANG, MINGHAI WEI, JIAN YIN

**Affiliations:** Department of Neurosurgery, The Second Affiliated Hospital of Dalian Medical University, Dalian, Liaoning 116044, P.R. China

**Keywords:** epilepsy, triple pathology, hippocampal sclerosis, focal cortical dysplasia, ganglioglioma

## Abstract

The coexistence of three intracranial lesions related to epileptic pathogenesis is known as ‘triple pathology’ and has rarely been reported. In this study we report a case of temporal lobe epilepsy (TLE) with the coexistence of hippocampal sclerosis (HS), focal cortical dysplasia (FCD) and ganglioglioma in the temporal lobe. A 29-year-old male who had experienced recurrent seizures for four years was admitted to hospital. Cerebral magnetic resonance imaging (MRI) was conducted and T2-weighted and fluid-attenuated inversion recovery sequence (FLAIR) images revealed a reduced hippocampal volume with an increased FLAIR signal on the right side and a slightly enlarged temporal horn, which are typical imaging findings for HS and FCD. The patient underwent resectioning of the right anterior temporal lobe, hippocampus and amygdala, in addition to the lesion located in the medial temporal lobe. Immunohistochemical analysis of the medial temporal lobe lesion confirmed a ganglioglioma (WHO grade I) in the medial temporal lobe. During the first eight months following surgery, the patient's seizures were controlled with zonisamide and phenytoin. Electroencephalogram (EEG) assessment post-surgery confirmed the absence of epileptic discharges. Based on a literature review and a detailed review of this case, we postulate two possible explanations for the pathogenesis of ‘triple pathology’: i) ‘triple pathology’ is a combination of pathological progression and occasionality; and ii) ‘triple pathology’ lesions have similar pathological origins.

## Introduction

Temporal lobe epilepsy (TLE) is the most common form of partial epilepsy in humans and is generally resistant to treatment (1). A randomized clinical trial has demonstrated that surgery is superior to prolonged medical therapy for TLE patients (2). However, surgical treatments have failed to provide a seizure-free outcome in 20–30% of TLE patients (3). Several potential explanations for the surgical treatment failures in TLE have been proposed. Insufficient resectioning of the mesial temporal structures may be a major cause of seizure recurrences following epilepsy surgeries (4,5). Recent studies have indicated that the coexistence of mesial TLE with hippo-campal sclerosis (HS) and a temporal neocortical lesion (so called ‘dual pathology’) may be an important cause of surgical failures in patients with TLE (6). Common neocortical lesions include focal cortical dysplasia (FCD), vascular malformations (which include arteriovenous malformations, aneurysms and cavernomas) and benign primary brain tumors (which include gangliogliomas, dysembryoplastic neuroepithelial tumors, pleomorphic xanthoastrocytomas and low-grade astrocytomas) (7). ‘Triple pathology’ in TLE refers to the coexistence of HS with two other intracranial lesions related to the pathogenesis of epilepsy (8). TLE patients with ‘triple pathology’ have rarely been reported. In this study, we report a rare case of the coexistence of HS, FCD and ganglioglioma in the mesial temporal lobe in TLE patients with ‘triple pathology.’ The possible pathogenesis of ‘triple pathology’ in epilepsy is discussed. This study was approved by the Ethics Committee of the Second Affiliated Hospital of Dalian Medical University. Written informed consent was obtained from the patients and the procedures were approved by institutional review boards.

## Case report

A 29-year-old right-handed male had experienced recurrent seizures for four years. The patient's seizures began with a paroxysmal disturbance of consciousness, followed by automatic movements such as swallowing, smacking of the lips or glazed eyes and progressed to flexing of the limbs, shaking and stiffening. Seizures occasionally occurred without an aura and lasted from several seconds to minutes. Initially, the patient's seizures only occurred three to four times a year, but this frequency gradually increased to nearly once a week. The patient was born prematurely and had no history of febrile convulsions, meningitis or encephalitis. The patient had no history of other medical conditions and the findings of the neurological examination were normal. The patient had been treated with various drugs including sodium valproate, carbamazepine, zonisamide, clobazam, and Chinese herbal medicine. However, the patient's seizures became medically intractable prior to being admitted to The Epilepsy Center of The Second Affiliated Hospital of Dalian Medical University.

**Figure 1.**
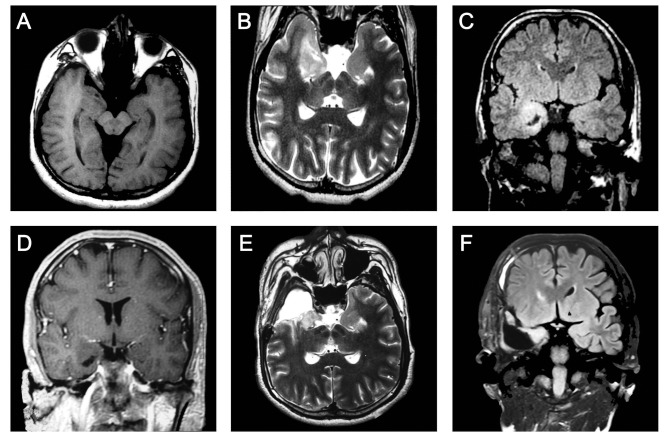
(A and B) Pre-surgery T1- and T2-weighted images show an approximate 2.0×1.4 cm region of abnormal signal in the right anterior and medial temporal lobe (long T1 and T2 weighted signal). (C) T2-weighted and FLAIR images showed a reduced hippocampal volume with relatively increased signal on the right side and slightly enlarged temporal horn, which are typical findings of HS and FCD. (D) Contrast-enhanced MRI showed no signs of enhanced signals. (E and F) Post-surgery T1- and T2-weighted images showed partial lesion residual in right temporal lobe. HS, hippocampal sclerosis; FCD, focal cortical dysplasia.

Cerebral magnetic resonance imaging (MRI) revealed a ∼2.0×1.4 cm region of abnormal signal in the right anterior and medial temporal lobes (long T1- and T2-weighted signal). T2-weighted and fluid-attenuated inversion recovery sequence (FLAIR) images revealed a reduced hippocampal volume with an increased FLAIR signal on the right side and a slightly enlarged temporal horn, typical in patients with HS and FCD. Focal cortical thickening with subcortical hyperintensity was noted in the right frontal lobe. These findings indicated the coexistence of FCD in the frontal lobe ipsilateral to the side of HS (Fig. 1A–C). Contrast-enhanced MRI revealed no sign of enhanced signals (Fig. 1D).

**Figure 2.**
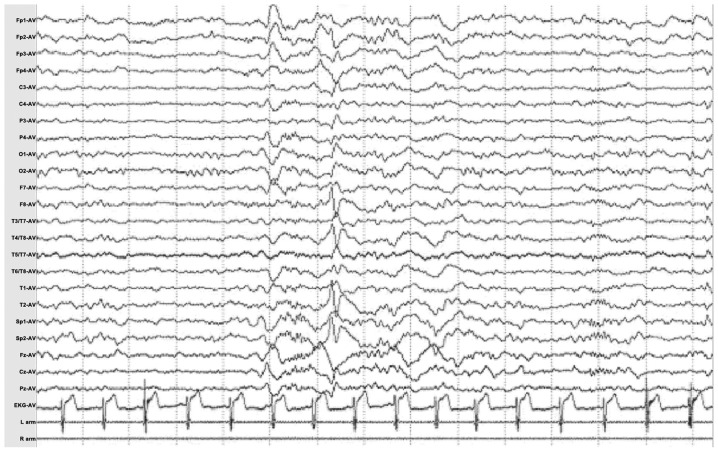
Pre-surgery electroencephalogram (EEG) revealed multiple continuous slowing and intermittent epileptiform discharges from the right anterior temporal region, T2, Sp2 and spreading to the ipsilateral frontal lobe.

Video-electroencephalography (video-EEG) was used to monitor one episode of a seizure attach. The attack was observed to involve abrupt disturbances in consciousness during the daytime, followed by left upper limb stiffening, head tilting to the left side, blinking and swallowing movements; lasting for a total of 70 sec. The interictal EEG indicated multiple continuous slowing and intermittent epileptiform discharges from the right anterior temporal region, T2 and Sp2, spreading to the ipsilateral frontal lobe. The ictal EEG demonstrated 3–4.5 Hz slow waves rising from the right anterior temporal lobe, then charged_6–7 Hz sharp waves in the overall right temporal area, gradually spreading to the right hemisphere as continuous 3 Hz slowing spikes (Fig. 2).

Due to the coexistence of HS and FCD, we predicted a poor outcome for treatment with medication. Following complete pre-surgical assessment, the patient underwent resectioning of the right anterior temporal lobe, hippocampus and amygdala, in addition to the lesion located in the medial temporal lobe. According to the surgeon, the hippocampus, amygdala and anterior temporal lobe felt solid, particularly the medial temporal lobe lesion, which was located adjacent to the thalamus and basal ganglia. We failed to achieve a complete resection of the medial temporal lobe lesion due to its anatomical location; however, the hippocampus, amygdala and anterior temporal lobe cortex were excised successfully (Fig. 1E and F). Pathological examination of the excised tissue revealed hippocampal sclerosis with neuronal cell loss accompanied by astrogliosis.

**Figure 3.**
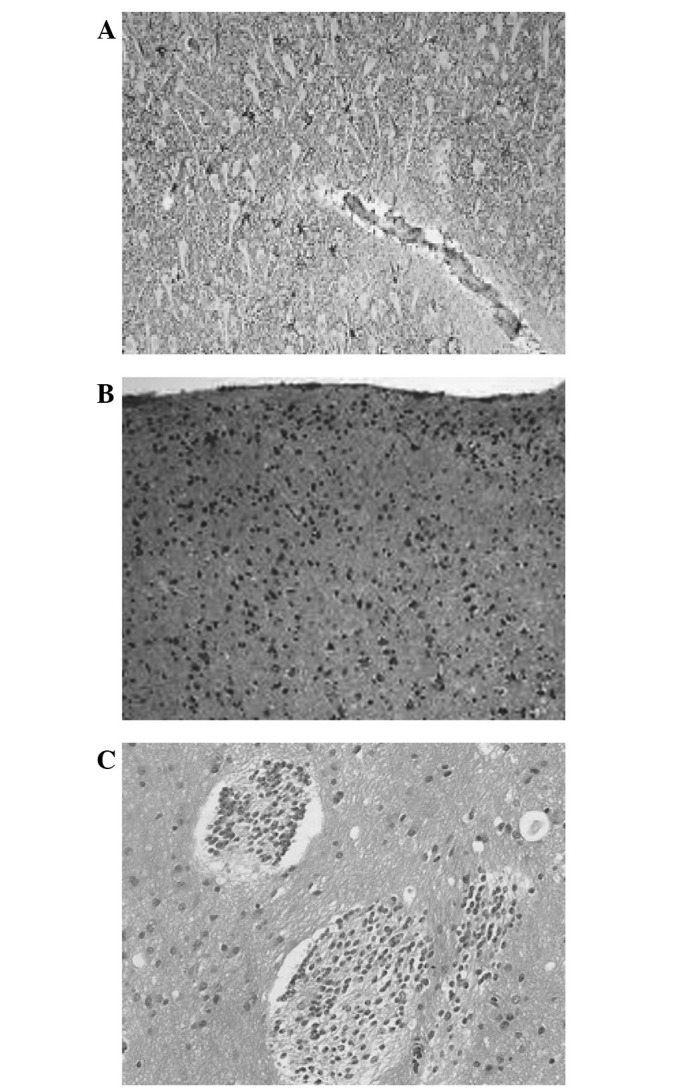
Histopathological examination of surgical specimens. (A) Hippocampal sclerosis; (B) focal cortical dysplasia; (C) ganglioglioma.

Histopathological examination confirmed FCD (type IIA) of the anterior temporal lobe with dysmorphic neurons and a malformation of the cortical structure. Immunohistochemical findings revealed disorganized neurons, glial fibrillary acidic protein (GFAP), oligo-2 glial cells, a small quantity of CD34 and a Ki-67 index <1%. The medial temporal lobe lesion was confirmed as ganglioglioma (WHO grade I). Immunohistochemistry results for the lesion revealed that the tumor cells were positive for GFAP, NeuN and CD34 and had a Ki-67 index <1%. Following surgery, the patient was seizure-free for eight months (Fig. 3). During the first eight months following surgery, the patient's seizures were controlled with zonisamide and phenytoin. An EEG assessment eight months post-surgery confirmed that there had been no epileptic discharges or relapses.

## Discussion

The coexistence of three intracranial lesions related to epileptic pathogenesis is known as ‘triple pathology’. The occurrence of ‘triple pathology’ in epilepsy patients was initially proposed by Maciunas *et al* (9) and Samura *et al* (10). Samura *et al* reported a case of TLE with the coexistence of HS, FCD and cavernoma (CA) (10). The authors theorized that FCD was the epileptic lesion responsible for secondary hippocampal damage and gradually induced intractable epilepsy, while the existence of a CA was not an aggravating factor. Thus, they conducted a resection of the right anterior temporal lobe, hippocampus and amygdala, in addition to a biopsy on the FCD lesion; no treatment for CA was performed. The patient achieved a 12-month seizure-free outcome, which preliminarily fitted with their assumption. Maciunas *et al* reported two cases of TLE with ‘triple pathology’: one case had CA, FCD and HS, while the other had CA, FCD and venous angioma (9). By reviewing their cases, the authors concluded that FCD is heterogeneous with various lesions appearing to coexist with other pathologies.

FCD has been reported with a high frequency of coexistence with other pathological lesions in TLE (11). Recent studies have indicated that FCD has molecular similarities to tuberous sclerosis, ganglioglioma and hemimegalencephaly (12). In the current study, we demonstrated that FCD and ganglioglioma were different histological expressions due to the same pathogenesis, that is, heteromorphism, causing recurrent seizures, which gradually induced hippocampal damage or HS. Therefore, we conclude that ‘triple pathology’ lesions may have similar pathological origins.

A ganglioglioma is a neoplasm comprised of neurons and glial cells, which has a slow growth process, occasional anaplasia and an incidence rate of 0.4–4% among central nervous system (CNS) tumors (WHO grade I–II) (13). Although a ganglioglioma is not a major cause of refractory seizures, it is normally present in intractable epilepsy cases and considered to be a major cause of secondary epilepsy (14,15). Clinicians and researchers acknowledge that the chronic growth process of a ganglioglioma may stimulate and oppress its surrounding cortex, thereby inducing seizure attacks (16). In the current study we failed to perform a complete resection, but the patient had a successful outcome, which confirmed our two initial assumptions. Firstly, FCD and HS may be the lesions responsible for epilepsy while the ganglioglioma had only a minor role in the seizure network; therefore, the complete resection of HS and FCD would result in a good recovery. Secondly, the ganglioglioma induced seizures were likely to be due to the surrounding cortex. A surgical resection of the ganglioglioma was incomplete and certain surgical injuries were sustained to the surrounding cortex, which may have jeopardized the primary conduction network and led to the intractable seizures.

In conclusion, based on the literature review and a detailed review of this case, we postulate two possible explanations for the pathogenesis of ‘triple pathology’: i) ‘triple pathology’ is a combination of pathological progression; and ii) ‘triple pathology’ lesions have similar pathological origins.
